# Feasibility of Diagnosing Dead Regions Using Auditory Steady-State Responses to an Exponentially Amplitude Modulated Tone in Threshold Equalizing Notched Noise, Assessed Using Normal-Hearing Participants

**DOI:** 10.1177/23312165231173234

**Published:** 2023-06-29

**Authors:** Emanuele Perugia, Frederic Marmel, Karolina Kluk

**Affiliations:** Manchester Centre for Audiology and Deafness, School of Health Sciences, Faculty of Biology, Medicine and Health, University of Manchester, Manchester, UK

**Keywords:** auditory steady-state response, cochlear dead region, amplitude modulation masking, threshold equalizing noise

## Abstract

The aim of this study was to assess feasibility of using electrophysiological auditory steady-state response (ASSR) masking for detecting dead regions (DRs). Fifteen normally hearing adults were tested using behavioral and electrophysiological tasks. In the electrophysiological task, ASSRs were recorded to a 2 kHz exponentially amplitude-modulated tone (AM2) presented within a notched threshold equalizing noise (TEN) whose center frequency (CF_NOTCH_) varied. We hypothesized that, in the absence of DRs, ASSR amplitudes would be largest for CF_NOTCH_ at/or near the signal frequency. In the presence of a DR at the signal frequency, the largest ASSR amplitude would occur at a frequency (*f_max_*) far away from the signal frequency. The AM2 and the TEN were presented at 60 and 75 dB SPL, respectively. In the behavioral task, for the same maskers as above, the masker level at which an AM and a pure tone could just be distinguished, denoted AM2ML, was determined, for low (10 dB above absolute AM2 threshold) and high (60 dB SPL) signal levels. We also hypothesized that the value of *f_max_* would be similar for both techniques. The ASSR *f_max_* values obtained from grand average ASSR amplitudes, but not from individual amplitudes, were consistent with our hypotheses. The agreement between the behavioral *f_max_* and ASSR *f_max_* was poor. The within-session ASSR-amplitude repeatability was good for AM2 alone, but poor for AM2 in notched TEN. The ASSR-amplitude variability between and within participants seems to be a major roadblock to developing our approach into an effective DR detection method.

## Introduction

Presbycusis, that is, age-related hearing loss, leads to a gradual loss of sensory hair cells, particularly in the basal region of the cochlea (Schuknecht & Gacek, 1993), with a greater loss of outer hair cells (OHCs) than inner hair cells (IHCs) and spiral ganglion cells (SGCs) as a function of age (Makary et al., 2011; Schuknecht & Gacek, 1993; Wu et al., 2019). It is assumed that the absence of OHCs alone leads to an elevation of hearing thresholds at high frequencies of about 50 to 60 dB HL (Schmiedt, 2010), whereas a loss or degeneration of both OHCs and IHCs can lead to a more severe hearing loss (Moore & Glasberg, 2004; Schmiedt, 2010). Near or complete loss of IHCs and/or SGCs in a given region along the basilar membrane (BM) in the cochlea is referred to as a cochlear dead region (DR; Moore et al., 2000). In such a region, the IHCs and/or spiral ganglion neurons function so poorly that information about BM vibrations in this region is not transmitted up the auditory pathway (Moore, 2004; Moore et al., 2000). However, a tone that produces maximum BM vibration within a DR may still be detected via off-place (or off-frequency) listening (Moore, 2004; Moore et al., 2000). In order to be detected via off-place IHCs, the tone needs to be presented at a level higher than normal to evoke BM vibration sufficient for its detection away from the point of maximum BM displacement (Moore, 2004; Moore et al., 2000).

The extent of a DR is defined in terms of its edge frequencies (Kluk & Moore, 2006a; Moore et al., 2000), which can be estimated using masking techniques such as psychophysical tuning curves (PTC) and the threshold equalizing noise (TEN) test (Kluk & Moore, 2006b; Moore et al., 2000).

PTCs can be measured using fixed masker frequencies (Chistovich, 1957; Kluk & Moore, 2005) or a sweeping masker frequency (Sęk et al., 2005), usually using simultaneous masking of a pure tone signal that is fixed in level and frequency. The masker is usually a narrow-band noise whose level and center frequency are adjusted (Kluk & Moore, 2006b). The purpose of the test is to identify the masker center frequency at which the level of the masker required to mask the signal is lowest. For normally hearing participants, and participants with hearing loss but without DRs, the frequency at which the masker level is lowest (also known as tip of the PTC) lies close to the signal frequency. For hearing-impaired participants with a DR, the tip of the PTC is shifted away from the signal frequency. The frequency at the tip indicates the edge frequency of the DR. Another behavioral method of detecting DRs is via the TEN test, which involves measurement of the masked threshold of a pure tone in a broad-band spectrally shaped TEN. The level of the TEN is fixed while the level of the tone is adjusted to find the masked threshold. The TEN is designed to produce almost equal masked thresholds across a wide frequency range for normal-hearing listeners (Moore et al., 2000, 2004). The TEN test is a clinical tool with clear criteria for diagnosing a DR at a given frequency. A DR is deemed to be present at the test frequency if the masked threshold is ≥ 10 dB above the TEN level and ≥ 10 dB above the absolute threshold at the test frequency. Comparisons of the PTC and TEN test techniques and their advantages and disadvantages are discussed in detail in the literature (Kluk & Moore, 2006b; Moore et al., 2000; Pepler et al., 2014; Warnaar & Dreschler, 2012). One disadvantage of these tests is that both require participants’ cooperation and some training to obtain stable results. Therefore, they are not suitable for use with young children and with adults who are unable to give behavioral responses.

To overcome these limitations, electrophysiological approaches have been proposed, for example using either auditory steady-state responses (ASSRs) or the acoustic change complex (ACC). ASSRs are potentials evoked by a periodically modulated signal, and have the same periodicity as the modulator (Picton et al., 2003a). The modulation rate determines which neural generators contribute to the ASSRs: a modulation rate of about 80 Hz elicits mainly subcortical responses whereas a modulation rate of about 40 Hz elicits mainly cortical responses (Luke et al., 2017; Rance, 2008). The ACC is a cortical potential elicited by a change during an ongoing acoustic stimulus (Martin & Boothroyd, 1999, 2000).

Markessis et al. (2009) used ASSRs to measure tuning curves and compared these electrophysiological tuning curves (ETCs) to PTCs (measured using 2 kHz pure tones), for six normally hearing adults. The ETCs were measured for a 2 kHz amplitude-modulated (AM) signal (probe) set at a level corresponding to the individual's ASSR threshold when presented simultaneously with 1 of 13 narrow-band maskers of different center frequencies. The PTCs were measured for pure tone signals set to 10 dB SL. For both techniques, the levels of the maskers were adjusted. The masked ASSR threshold was defined by the minimum masker level required to fully suppress the ASSR (i.e., a nonsignificant result of squared phase coherence tests). The ETCs were broader than the PTCs. The tips were more often, albeit not significantly, shifted upward in frequency relative to the signal frequency for the ETCs than for the PTCs. Markessis et al. (2009) associated this with either two-tone inhibition or suppression effects. Despite the reasonable similarity between the PTCs and ETCs, the latter required an average of 15 min for each of the 13 masker center frequencies and required high-level maskers for masker center frequencies away from the signal frequency. The masker levels required for participants with moderate or severe hearing loss might be too high to be safe, and the long duration of the protocol reduces the feasibility of using ETCs as a clinical method.

Wilding et al. (2011) proposed a faster method that required a lower masker level. Instead of measuring ASSR thresholds in the presence of several maskers, Response Amplitude Curves were derived from ASSR amplitudes collected for 2 kHz AM tones presented with a sweeping narrow-band masking noise. The masker center frequency was continuously varied between 1 and 4 kHz (in both upward and downward directions). The level of the signal and the masker were both 50 dB above the participants’ ASSR threshold for the test frequency (signal-to-noise ratio, SNR  =  0 dB). The test took approximately 32 min. The mean estimated Response Amplitude Curve tip frequency, that is, the masker center frequency that gave the minimum ASSR amplitude, was 2250 Hz. The authors quantified the reliability of the test by calculating the coefficient of reliability, which is defined as twice the standard deviation of the differences between two separate sessions (Bland & Altman, 1986). The value was 342 Hz for the 2 kHz signal frequency. The authors attributed the upward tip-shift to a basal spread of excitation due to the high stimulus levels.

Recently, the ACC threshold was proposed as a way of diagnosing DRs (Kang et al., 2018). Similar to the TEN test, the masker was a TEN played at a fixed level of 60 dB/ERB_N_ (ERB_N_ is the average value of the Equivalent Rectangular Bandwidth of the auditory filter at moderate sound level for young listeners with no known hearing defects; centered at 1 kHz  =  132 Hz; Glasberg & Moore, 1990) while the level of a pure tone was adjusted to find the ACC masked threshold, that is, the lowest level at which the ACC was present. Kang et al. (2018) found that normally hearing participants and participants with a hearing loss without DRs had an average ACC masked threshold lower than 12 dB SNR; hence, they proposed this value as the equivalent of the behavioral TEN test criterion. However, the unclear inclusion criteria (e.g., no information was given about individual audiograms, or, critically, at which frequencies DRs were present) and the lack of systematic investigation of ACC thresholds both in quiet and in TEN (i.e., the participants were not always tested at the same frequencies), make it difficult to gauge the potential of this approach as a clinical tool.

The abovementioned techniques had a common drawback: they searched for minimum electrophysiological responses, which mean that the responses of interest may have been obscured by the electroencephalogram (EEG) noise floor. Detection of ASSRs close to the EEG noise floor is especially problematic, as there is no consensus as to the best stopping criterion (D’haenens et al., 2010; Luts et al., 2008; Wilding et al., 2012), or the best statistical method for avoiding an inflated type I error rate (Cebulla et al., 2001, 2006; Dobie & Wilson, 1996; Stürzebecher et al., 1999, 2005; Valdes et al., 1997). To avoid these problems, we propose an approach where the search is for a maximum electrophysiological response instead of a minimum.

In Markessis et al. (2009) and Wilding et al. (2011), the ASSR amplitude decreased as the frequency of a narrow-band masker approached the frequency of the ASSR-evoking signal. In the present study, an AM2 tone of fixed frequency and level was presented in the presence of a TEN with a spectral notch, called here “notched-TEN” (the term “full TEN” refers to the TEN without a notch). The center frequency of the notch (CF_NOTCH_) moved toward the signal frequency so that the ASSR amplitude gradually increased as CF_NOTCH_ approached the signal frequency, reaching a maximum at/or near the signal frequency, and the ASSR amplitude gradually decreased as the CF_NOTCH_ moved above/below the signal frequency. We hypothesized that the value of CF_NOTCH_ at which the ASSR amplitude was highest would correspond to the edge frequency of a DR and thus could be used as an objective way for diagnosing DRs. Specifically, our hypotheses were that for normally hearing participants and participants with hearing loss without DRs, the largest ASSR amplitude would be recorded for CF_NOTCH_ centered on/or near the signal frequency. For participants with DRs encompassing the signal frequency, the largest ASSR amplitude would occur for CF_NOTCH_ away from the signal frequency and close to the edge frequency of the DR. In the current study, we tested this proposed approach using normally hearing adults.

A pioneering study using notched-noise and an electrophysiological measure was performed by Picton et al. (1979). In a series of experiments, tone-pips were presented with notched white noise (2-octave-wide notch) and Auditory Brainstem Responses were used to estimate frequency-specific auditory thresholds. In a pilot phase of the current study, we sought the minimum notch width required to obtain a significant difference in ASSR amplitude between two conditions: AM2 in full TEN and AM2 in notched TEN with CF_NOTCH_ at 2 kHz. We found that a width of at least 10 ERB_N_ (i.e., 2406 Hz) was required. Thus, following Picton et al. (1979), we decided that a 2-octave width was also appropriate for our ASSR method.

Wilding et al. (2011) showed that ASSRs recorded to sounds presented at different SPLs but the same SLs (50 dB above individual participant's ASSR threshold) lead to heterogeneous responses across participants. Thus, in the current study, the ASSR stimuli were presented at the same fixed level in dB SPL for all participants.

Using a similar notched-TEN technique, the masker level at which an AM and a pure tone carrier could just be distinguished, denoted AM2ML, was determined, for low (the same SL across all participants) and high signal levels similar to those used in our ASSR procedure (the same SPL across all participants). We further hypothesized that each participant would have *f_max_* at the same/similar notch position for the psychophysical and ASSR measurements. The relatively large notch (2-octave wide) may result in some inter-participant variability, but we expected this variability to be participant specific and thus remain the same for the two techniques, leading to similar *f_max_* for the psychophysical and ASSR measurements.

Previous ASSR studies have assessed the test–retest reliability of ASSR amplitudes and raised concerns about their variability and statistical significance (D’haenens et al., 2008; Wilding et al., 2012). To evaluate within-session reliability, we recorded ASSRs within two half-tracks: one half-track with the notch moving upwards in frequency, and one half-track with the notch moving downwards in frequency.

The agreement between the psychophysical and ASSR methods, and the within-session ASSR amplitude repeatability were used to evaluate the feasibility of using the ASSR notched-noise technique to diagnose DRs.

## Materials

### Participants

Fifteen adults (8 females) with a mean age of 23.4 years and a range of 18 to 32 years participated. All participants had audiometric thresholds at or below 20 dB HL for frequencies from 0.5 to 8 kHz and differences between ears were ≤15 dB (British Society of Audiology, 2018). The participants reported no history of neurological or psychiatric disorders.

This study was approved by the University of Manchester Research Ethics Committee (Ref 16365) and informed written consent was obtained from all participants after they were informed about the nature of the study. All participants were paid for their time.

### Stimuli

**Figure 1.**
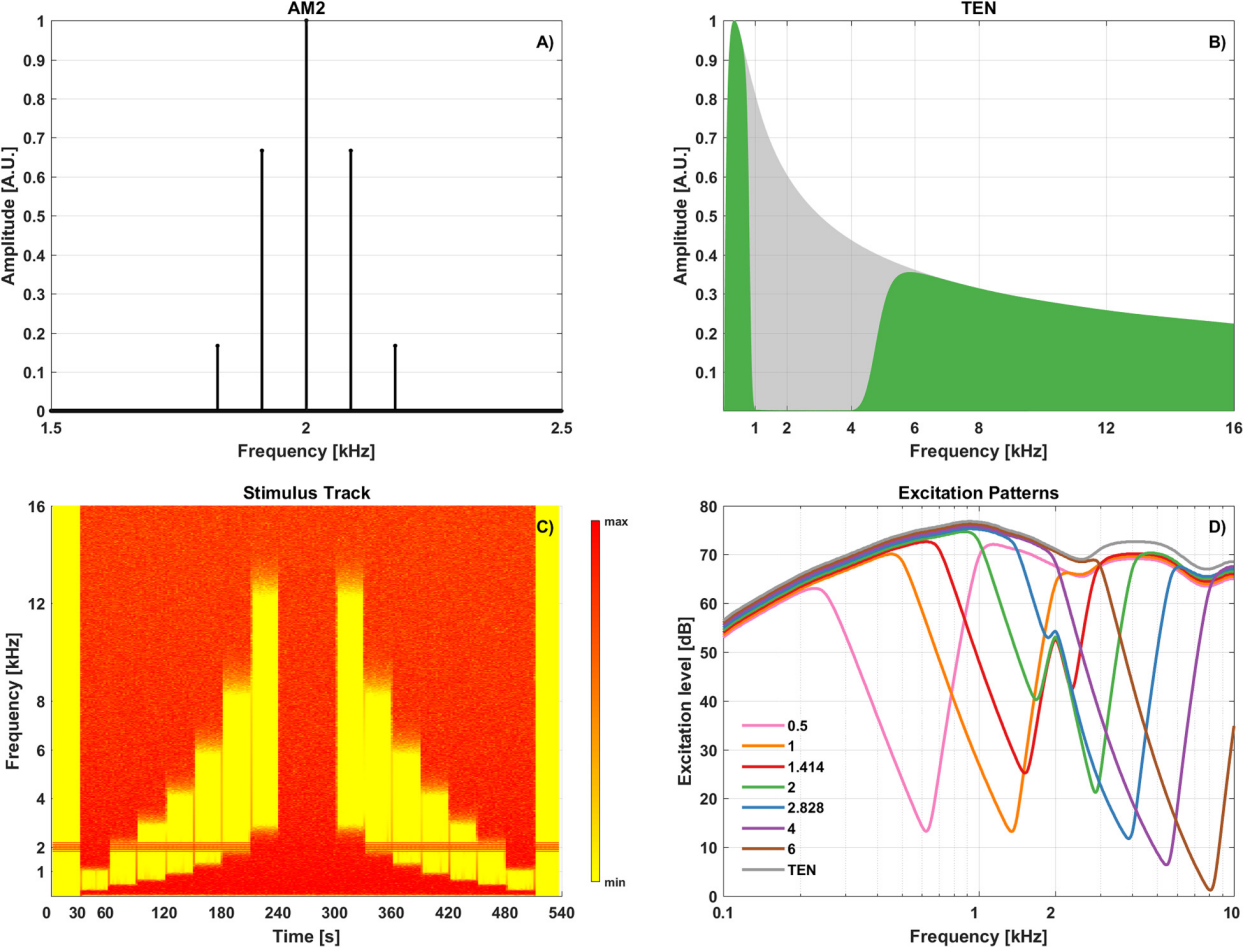
Stimuli and their excitation patterns. (A) Spectrum of the exponentially AM2. (B) TEN spectrum for a 2-octave-wide notch centered at 2 kHz (in green) and the full TEN (shaded in gray). (C) ASSR track with AM2 tone and TEN filtered by a moving notch (30s steps) in the time-frequency domain; the AM2 was presented alone, simultaneously with notched TEN with the notch centered at 0.5, 1, 1.41, 2, 2.82, 4, and 6 kHz, and with full TEN. (D) The estimated excitation patterns of the notched TEN and full TEN. AM2: amplitude-modulated target tone; ASSR: auditory steady-state response; TEN: threshold equalizing noise.

The stimuli were an exponentially amplitude modulated target tone (AM2, Figure 1A) and a TEN (Figure 1B; Moore et al., 2000, 2012).

The equation describing the AM2 stimulus (John et al., 2002) is:

$$s(t)=a*sin(2\pi f_{c})*[2m_{a}({\left((1+sin(2\pi f_{m}))/2\right)}^{N}-0.5)+1]$$

where *a* is the amplitude of the signal, *m_a_* the modulation depth (100% here), *f_c_* the carrier frequency (2 kHz) and *f_m_* the modulation frequency (87 Hz). The power of the exponential envelope *N* was 2.

The use of an exponential envelope increased the “sharpness” of the modulation, hence increasing ASSR amplitude. A potential drawback of exponential envelope tones is that their bandwidth increases as the exponent increases, which poses a risk of the stimulus losing frequency specificity. To minimize that risk we chose N  =  2 (Eggermont, 1994; John et al., 2002). The modulation frequency of 87 Hz meant that the ASSR originated mainly from the level of the brainstem (Luke et al., 2017; Rance, 2008; Weisz & Lithari, 2017). The carrier frequency of 2 kHz was chosen to allow comparisons of the results with those for other studies (Markessis et al., 2009; Wilding et al., 2011).

The TEN used in the present study was spectrally shaped to produce equal masked thresholds in dB SPL between 50 and 16,000 Hz. Like other TEN versions (Moore et al., 2004, 2012), it was implemented using Pumplin’s “low-noise” technique (Pumplin, 1985). The TEN was filtered to produce a 2-octave-wide spectral notch centered on a log scale at 0.5, 1, 1.41, 2, 2.82, 4, or 6 kHz (CF_NOTCH_). The filter was a 5th order Chebyshev type II, which gave a minimum attenuation in the notch of 30 dB.

All stimuli were generated using a personal computer (HP with dual processor Intel i7 6700 at 3.40 GHz, RAM of 32 GB and Windows 7) and MATLAB (R2013a, 8.1.604, 64-bit) and were presented via a Focusrite Scarlett 2i2 USB soundcard at a sampling rate of 44.1 kHz and a resolution of 24 bits. Stimuli were presented through Etymotic Research (ER) 2 Insert Earphones, which have a flat frequency response (±2 dB) at the eardrum for frequencies up to 10 kHz.

Stimulus levels at the output of the ER2 Insert Earphones were calibrated using a Brüel and Kjær type 2250 (Brüel and Kjær, Nærum, Denmark) sound-level meter in A-weighted Leq mode in combination with a GRAS IEC711 Coupler (RA0045, G.R.A.S. Sound and Vibration A/S, Holte, Denmark). Although the TEN version used in the present study has not been used previously, the standard TEN principle (i.e., equal masked thresholds produced for normal-hearing participants) holds, given the flat response of the ER2 Insert Earphones (Moore et al., 2000, 2004, 2012).

### Excitation Patterns

The excitation patterns elicited by the stimuli were estimated using the code and model proposed by Moore et al. (1997). This allowed us to choose the order and ripple of the filter used to create the notches in the TEN. The excitation patterns evoked by AM2 presented simultaneously with each of the seven notched TEN stimuli and with the full TEN are shown in Figure 1D. The TEN was predicted to be least effective as a masker when the notch was centered at 1.41 (in red), 2 (in green), and 2.82 (in blue) kHz.

### Procedure

Ear examination (otoscopy) and air-conduction pure tone threshold audiometry were carried out using the British Society of Audiology recommended procedures (British Society of Audiology, 2016, 2018). The test ear for the subsequent tasks was the one with the most constant (smallest variability across frequency) audiogram.

#### Psychophysical Task

The experimental procedure was implemented via custom MATLAB software (R2017a). The absolute threshold for the AM2 signal and AM2MLs were measured using an adaptive 2-alternative forced-choice (2AFC) task, with a two-down one-up procedure for threshold and a two-up one-down procedure for AM2ML, which track the 70.7% correct point on the psychometric function (Levitt, 1971). The two observation intervals were marked by a light flashing on a computer screen, and visual feedback was provided. There was a 50 ms silent interstimulus interval (ISI) and a 500 ms silent period between observation intervals. The step size was 4 dB for the first 4 turnpoints (changes in masker level direction) and 2 dB for the remaining 12 turnpoints. The mean and the standard deviation of the stimulus levels at threshold and at AM2MLs were calculated as the mean across the last 12 turnpoints. If the standard deviation across the last 12 turnpoints was more than 3 dB, the condition was repeated. The threshold and AM2MLs were calculated as the mean across two repetitions. Prior to testing, each participant performed three practice trials for both tasks.

For the absolute AM2 threshold measurements, a randomly chosen interval contained the AM2 stimulus, while the other interval was silent. The AM2 stimulus was a 230 ms (including 10 ms raised-cosine onset and offset ramps) exponentially AM tone with a carrier frequency of 2 kHz and a modulation rate of 86.95 Hz. The nominal 87 Hz modulation rate was adjusted to 86.95 Hz so that an integer number of cycles occurred within the stimulus (John & Picton, 2000). The stimulus level was initially set to 30 dB SPL. Participants were asked to identify which interval contained the stimulus by pressing a key on a computer keyboard.

For the AM2ML measurement, a randomly chosen interval contained the fixed-level AM2, while the other interval contained a 2 kHz unmodulated carrier (pure tone) with the same root-mean-square (RMS) level; both intervals also contained the TEN whose level was adjusted according to the participant's response. The duration of each signal was 230 ms, including 10 ms raised-cosine onset and offset ramps, while the TEN duration was 250 ms (with 10 ms raised-cosine ramps). The TEN started 10 ms before the signal and ended 10 ms after the signal. Eight TEN conditions were used: full TEN, and seven notched TENs with the CF_NOTCH_ at 0.5, 1, 1.41, 2, 2.82, 4, and 6 kHz. The order of the TEN conditions was random. Two levels of the signal were used: (1) low level, with each signal (AM2 and pure tone) presented at 10 dB above the participant's absolute AM2 threshold (10 dB SL referred to AM2 threshold) and the starting TEN level below the signal level (SNR  =  5 dB). (2) High level, with the signal (AM2 and pure tone) presented at 60 dB SPL, and starting TEN level of 75 dB SPL (SNR  =  −15 dB). Participants were asked to identify which interval contained the AM2 signal. The rationale for using the low SL signal was to ensure sharp tuning of the basilar membrane (Oxenham & Shera, 2003) and to be able to compare our results to those of Markessis et al. (2009). The high signal level was chosen to allow comparison with the ASSR method.

Each participant took part in at least 17 measures^1^: absolute AM2 threshold and 16 AM2MLs (with 8 TEN conditions for 2 signal levels). This part of the experiment took up to 60 min to complete.

#### ASSR Conditions

To collect the ASSRs, the stimulus conditions were concatenated in a 9 min track (see also the video in the Supplemental Material). The notch went upwards in frequency for the first half of the track and downwards for the second half. The nine stimulus conditions were: the AM2 presented alone, simultaneously with each of the seven notched TENs, and with the full TEN. Each stimulus condition lasted 30 s and was presented twice within the track, once in the upward half-track and once in the downward half-track. For the first half of the track, the stimulus conditions were concatenated in the following order: AM2 alone, the seven notched TEN conditions from the lowest CF_NOTCH_ to the highest, then the full TEN. For the second half of the track, the order was: the full TEN, the notched TEN conditions from highest to lowest CF_NOTCH_, then AM2 alone. The AM2 signal and the TEN were presented at 60 and 75 dB SPL, respectively (SNR  =  -15 dB). The choice of − 15 SB SNR was evaluated with excitation patterns as described earlier (Moore et al., 1997). The concatenation of the stimulus conditions was used to keep the participant's state of arousal constant and thus their ongoing EEG similar across all conditions.

### EEG Acquisition

Continuous EEG was obtained using a Biosemi ActiveTwo System (BioSemi B.V., Amsterdam, the Netherlands) with a sampling rate of 2048 Hz. Thirty-two active electrodes were placed following the 10 to 20 system (but Oz was replaced with Iz); the Common Mode Sense (CMS) and Driven-Right-Leg (DRL) electrodes were placed at the left and right of Cz, respectively (Metting van Rijn et al., 1990). Signagel Electrode gel (Parker Laboratories, Fairfield, USA) was used to obtain stable offset voltages of at least ± 40 mV measured between CMS and each active electrode.

Participants lay in a comfortable recliner in a double-wall soundproof booth with lights turned off. The participants were asked not to pay attention to the sounds and to try to sleep or relax and refrain from body movements.

### ASSR Data Analysis

EEG analysis was done offline with custom MATLAB software (R2017a). EEG signals were discarded if they were completely flat or had abnormally large amplitude. The reference was the average of signals for parietal and occipital electrodes (i.e., P7; P3; Pz; P4; P8; PO3; PO4; O1; Iz and O2), as ASSRs were not expected for these regions. The strongest ASSR responses can be recorded at Cz or Fz (John et al., 2003; Picton et al., 2009). Therefore, the average signal for these two electrodes was used to carry out the following analyses.

Linear trends were removed from the response every 4.5 min (half-way through the track) using the MATLAB function detrend. The response was bandpass filtered from 77 to 97 Hz (10 Hz above and below the modulation frequency) using a 5th-order Chebyshev Type II filter with ripple of 30 dB. The response was then subdivided into sweeps of about 5 s duration for a total of 48 sweeps for each of the nine conditions and two half-tracks. For each condition and half-track, 25% of the sweeps with the highest RMS values were assumed to contain artifacts and were rejected (John et al., 2001). The final number of sweeps was 72 per condition or, when analyzing per half-track, 36 per condition and half-track. Weighted averaging was performed over the remaining sweeps by weighting them by their variance (John et al., 2001). We used weighted averaging to allow easy comparison with published work. The ASSRs were analyzed in the frequency domain. Fast Fourier Transforms (FFTs) were estimated across both sweeps and averages with a frequency resolution of 0.2 Hz.

### Statistical Analysis of Psychophysical Task

Mixed-effects modeling (Baayen et al., 2008; Winter, 2013) of the relations between AM2MLs, TEN conditions, and signal levels was carried out. The fixed effects were the TEN conditions, the signal levels (low and high), and their interactions. The random effects were random intercepts for participants, and random intercepts among participants within both TEN conditions and signal levels. We accounted only for baseline difference in thresholds for the participants; we assumed that the effects of the TEN and signal levels were the same for all participants.

The analysis was performed in R 3.6 (R Core Team, 2020) using the package *lme4* (Baayen et al., 2008) and evaluated via lmerTest (Kuznetsova et al., 2017) and *performance* (Lüdecke et al., 2021) with Kenward-Roger approximation for degrees of freedom.

### Statistical Analysis of ASSRs

The ASSRs were evaluated graphically by drawing Response Amplitude Curves based on ASSR amplitudes estimated via FFT. Hotelling’s T2 and F-test for hidden periodicity were used to determine the statistical significance of the ASSRs against the background EEG noise (Cebulla et al., 2001; Picton et al., 2003b; Valdes et al., 1997; Vanheusden et al., 2019). Both tests were based on the real and imaginary parts of the FFT. Hotelling’s T2 is a multivariate generalization of Student’s t-test, and it was calculated over the sweeps. The F-test for hidden periodicity (F-test, for brevity) compared to the power of the response at the modulation frequency to the mean power across 60 bins (12 Hz) above and 60 bins below the modulation frequency, using the averaged sweeps. The aim of using both metrics was to compare their performance and accuracy as a possible clinical tool in the proposed method. The EEG noise floor for each condition and half-track was estimated using the permutation approach proposed by Prendergast et al. (2017). This analysis was performed in MATLAB (9.2, R2017a).

### Agreement Between Psychophysical and ASSR Methods

The agreement between CF_NOTCH_ for which AM2ML (psychophysical *f_max_*) was largest and CF_NOTCH_ for which ASSR-amplitude was largest (ASSR *f_max_*) was assessed using the Bland-Altman method (Bland & Altman, 1986). This was achieved by plotting the difference between the two measurements against their mean. The bias between the methods was estimated as the mean difference. The 95% of the differences lie within the so-called Limits of Agreement (LoA), which are estimated by adding (upper limit) or subtracting (lower limit) the standard deviation multiplied by 1.96 times the bias. The 95% confidence interval for the bias was also calculated.

Repeatability within the same ASSR recording was also evaluated via Bland-Altman plots using the upward and downward half-tracks. In particular, the ASSR *f_max_* values for the upward and downward half-tracks were compared, as well as the ASSR amplitudes elicited by the AM2 in the two half-tracks. By definition (Bland & Altman, 1986), the coefficient of repeatability is twice the standard deviation of the mean difference between the half-tracks (which is assumed to be zero), normalized by N, where N is the number of participants. Although the conditions (such as the AM2 or a notched TEN) in the upward and downward half-tracks were inevitably not exact repeats of the same measure, the comparison of the responses in the upward versus downward half-tracks gave a good approximation of the repeatability.

## Results

### Psychophysical Task

**Figure 2.**
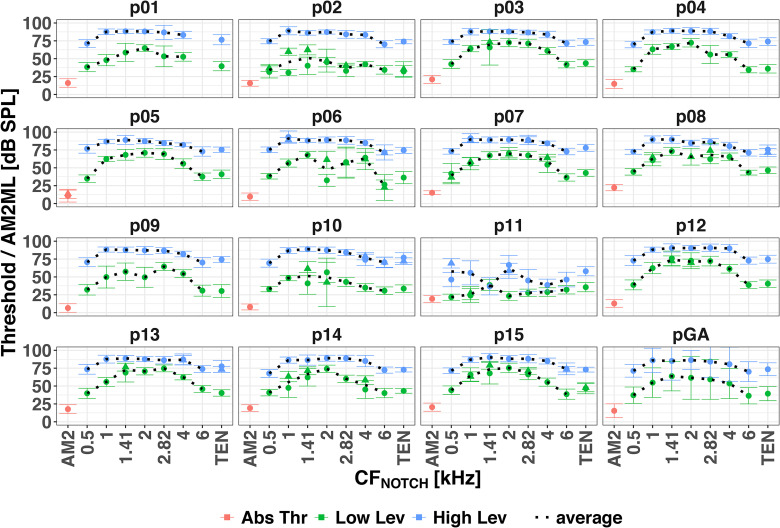
Psychoacoustical thresholds for each participant and the grand average over the participants (pGA). AM2 absolute thresholds in red, AM2MLs at the low level in green and at the high level in blue. Circles and triangles indicate the first and second repetition of a given condition (a second repetition was performed when the standard deviation of the first one was more than 3 dB). The average (black dotted line) was calculated across repetitions. Error bars show  ±  1 standard deviation of the levels at the last 12 turnpoints, or across the participants for the grand average. AM2: amplitude-modulated target tone.

Figure 2 shows the absolute AM2 thresholds in red, the AM2ML for the low signal level in green, and for the high signal level in blue for individual participants, and the grand average in black. Of the 15 participants, all but one (p11) showed the largest AM2ML when the CF_NOTCH_ was close to 2 kHz.

The mixed-effects model (conditional R^2^  =  0.95, marginal R^2^  =  0.77) showed significant main effects of TEN condition [F(7, 104.01)  =  85.55, *p* < .001] and signal level [F(1, 15)  =  385.92, *p* < .001], and a significant interaction [F(7, 104.03)  =  12.10, *p* < .001]. Pairwise comparisons with Bonferroni correction revealed that for the low signal level AM2MLs were significantly higher when the notch encompassed the AM2 frequency (i.e., CF_NOTCH_ at 1.41, 2, and 2.82 kHz) than when it did not. However, for the high signal level AM2MLs were significantly higher for CF_NOTCH_ at 1, 1.41, 2, and 2.82 kHz than at 0.5, 4 and 6 kHz, but there was no significant difference between AM2MLs with CF_NOTCH_ at 1, 1.41, 2, and 2.82 kHz, that is, the AM2ML curves for high signal level were flat between 1 and 2.82 kHz.

Overall, the AM2MLs were significantly higher for the high signal levels than for the low signal levels, and their dynamic range (i.e., the ratio between the maximum and minimum AM2MLs) was smaller for the high signal level than for the low signal level.

The psychophysical *f_max_* for the low signal level was at 1.41 (6 participants), 2 (7 participants) and 2.82 kHz (2 participants). For the high signal level, the peaks of the AM2ML curves were broad and flat (except for p11 who showed a double-peaked curve). It was not possible to establish individual *f_max_* for the high signal level AM2ML curves.

### ASSR

**Figure 3.**
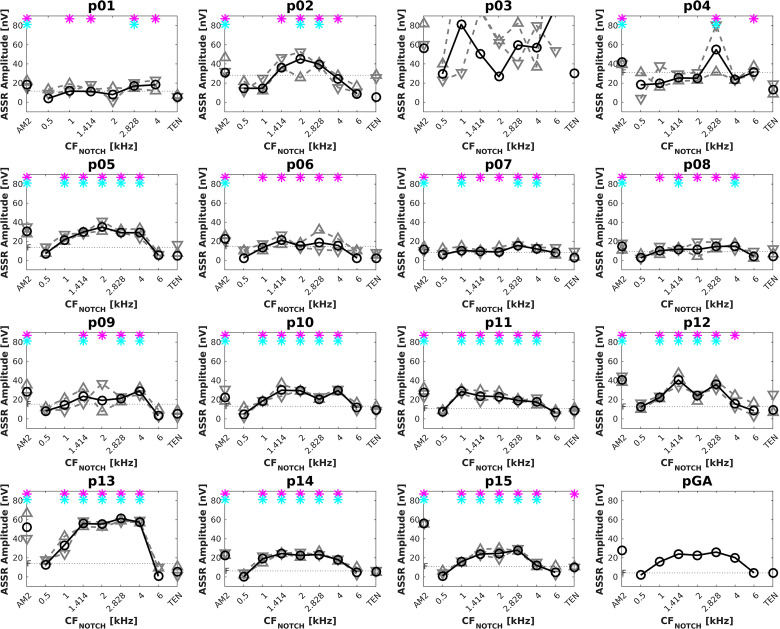
Individual ASSRs in response to the whole stimulus track (black circles and solid lines), upward half-track (gray upward-pointing triangles and dashed lines), and downward half-track (gray downward-pointing triangles and dashed lines) for each participant and the grand average over the participants (pGA, excluding measures for participant p03). The horizontal dotted line shows the EEG noise floor (F). The magenta and cyan asterisks indicated significant responses (*p* < .01) in the stimulus track via the F-test for hidden periodicity and Hotelling’s T2 test, respectively. ASSR: auditory steady-state response; EEG: electroencephalogram.

Figure 3 shows the ASSRs to the whole stimulus track (black circles and solid lines) and the ASSRs to the two half-tracks (gray triangles and dashed lines) separately for each participant and for the grand average across participants. ASSR amplitudes in the stimulus track indicated as significant (*p* < .01) by the F-test for hidden periodicity and/or the Hotelling’s T2 test are marked with magenta and/or cyan asterisks, respectively. The horizontal dotted line shows the EEG noise floor. The recording for participant p03 was corrupted, so their results were excluded from further analyses.

The stimulus track had the AM2 presented alone and simultaneously with the full TEN as two control conditions. Ideally, the former should have elicited a significant ASSR for each participant while the latter should have not. This was true for AM2 alone, as all participants showed significant responses according to both statistical tests. For the full TEN, all participants except p15 showed nonsignificant responses. Table 1 shows the conditions with significant ASSRs at *p* < .01 as determined using the two statistical tests. The ASSR amplitudes were significant for both tests in 60 cases out of 125, and only for the F-test in 19 cases.

**Table 1.** Number of Significant Responses for Each Condition Using the F-Test for Hidden Periodicity and Hotelling's T2 Test.

	AM2 alone	Notch center frequencies							TEN full
		0.5	1	1.41	2	2.82	4	6	
F-test	14	0	11	13	12	14	13	1	1
Hotelling's T2 test	14	0	8	9	8	12	9	0	0

AM2: amplitude-modulated tone; TEN: threshold equalizing noise.

The ASSRs for the notched TEN conditions were heterogeneous. Only a few participants showed a clear increase of the ASSR response as the notch approached 2 kHz. Participants p02, p05, p13, and p14 showed the predicted response pattern and the ASSR amplitudes for the grand average were close to the predicted pattern.

The maximum ASSR amplitudes among the notched TEN conditions occurred at CF_NOTCH_ of 1 (1 participant), 1.41 (4 participants), 2 (2 participants), 2.82 (4 participants), and 4 kHz (2 participants). The *f_max_* values varied more for the ASSR than for the psychophysical task.

Of interest were the ASSR amplitudes for AM2 alone and for the condition with the notched TEN with CF_NOTCH_ at 2 kHz. For AM2 alone, the mean (and range) ASSR amplitude was 30 (12–56) nV; while for the notched TEN with CF_NOTCH_ at 2 kHz the ASSR amplitude was 25 (8–55) nV. The two ASSR amplitudes did not differ significantly (Wilcoxon signed rank test: V  =  78, *p*  =  .11), confirming that, the ASSRs were not masked when CF_NOTCH_ was 2 kHz.

The average (and range) noise floor was 14.3 (6–31) nV. The ASSR amplitudes for AM2 alone and the full TEN were always above and below the noise floor, respectively. Note that for participant p15, despite their ASSR amplitude in the full TEN condition being below the noise floor (10.37 vs. 11.04 nV), the F-test was significant. The ASSR amplitude in response to the notched TEN with CF_NOTCH_ at 2 kHz was below the noise floor for two participants (p01 and p04) and these ASSR responses were not significant.

### Agreement Between Psychophysical and ASSR Data

**Figure 4.**
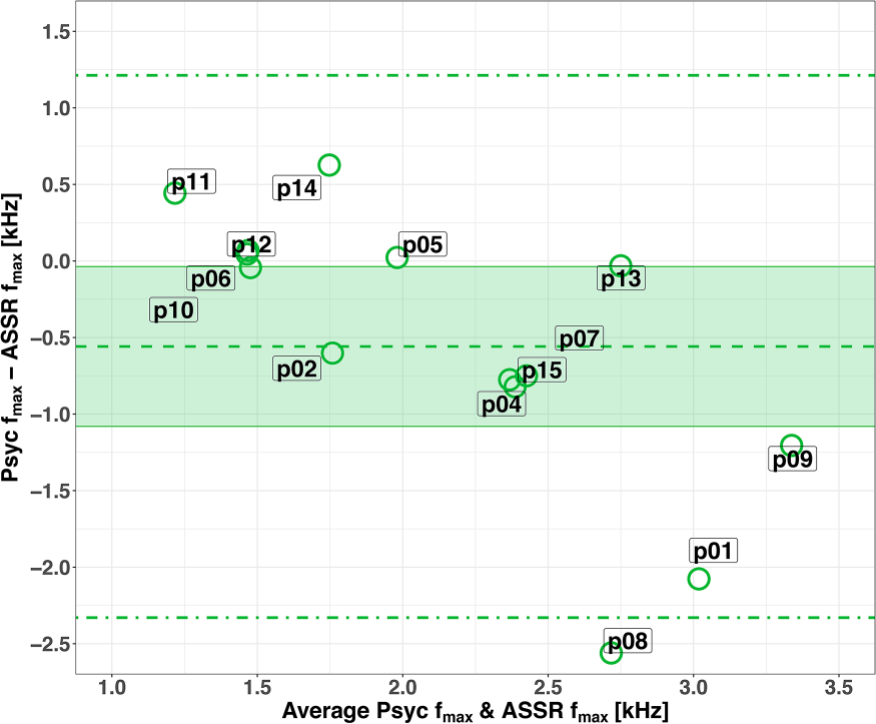
Agreement in *f_max_* between AM2ML for the low signal level and ASSR curves for each participant. ASSR: auditory steady-state response.

Five participants (p05, p06, p10, p12, and p13) had the same *f_max_* for the low signal level psychophysical task and the ASSR (high signal level). Due to the broad and flat peaks of the psychophysical AM2ML curves for the high signal level, it was not possible to compare ASSR *f_max_* with psychophysical *f_max_* for the high signal level. The Bland-Altman plots in Figure 4 illustrate the degree of agreement of *f_max_* between the psychophysical (for low signal level) and ASSR methods. The dashed and dot-dash lines show the bias and the LoA between the two methods; the shaded areas show the 95% confidence interval for the biases.

For the low signal level (Figure 4), the bias was −0.56 kHz, and the upper and lower LoA were 1.21 and −2.33 kHz. Considering that at 2 kHz the value of ERB_N_ is 240.6 Hz and the ERB_N_-number is 21.2 Cam (Moore, 2012, 2014), the values of the LoA expressed as ERB_N_-number were 17.1 and −22.4 Cam.

### Within-Participant ASSR Repeatability

Individual ASSRs for upward and downward half-tracks are shown in Figure 3 as gray upward-pointing and downward-pointing triangles and dashed lines. For the upward half-track, maxima of the ASSR amplitudes were observed for CF_NOTCH_ positions between 1 and 6 kHz. The majority of participants had the largest ASSR amplitudes for CF_NOTCH_ at 2.82 (5 participants) or 4 kHz (3 participants). For the downward half-track, the maxima fell at CF_NOTCH_ positions between 1 and 4 kHz, and the majority of participants had their maxima at 2 or 2.82 kHz (4 participants for each). The ASSRs for the downward half-track were more consistent and in line with the hypothesis (i.e., largest ASSR amplitudes when CF_NOTCH_ was at/or near the signal frequency) than those for the upward half-track. Only three participants showed maxima at the same CF_NOTCH_ position for the two half-tracks, p07, p11, and p12, and they had maxima with the TEN CF_NOTCH_ centered at 2.82, 1, and 1.41 kHz, respectively.

**Figure 5.**
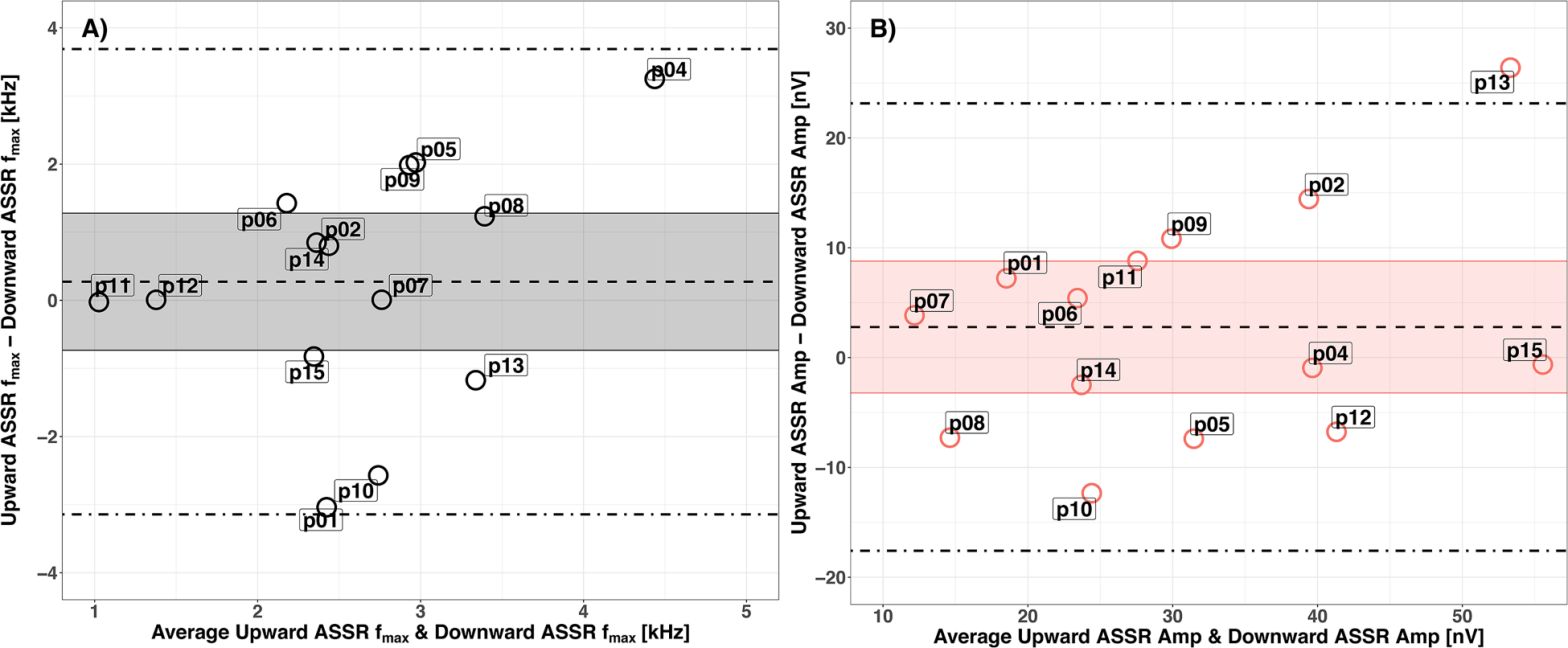
(A) Repeatability of ASSR *f_max_* between the upward and downward half-tracks, in kHz. (B) Repeatability of ASSR amplitudes for AM2 alone between the upward and downward half-tracks, in nV. AM2: amplitude-modulated target tone; ASSR: auditory steady-state response.

The estimated coefficient of repeatability between the upward and downward ASSR *f_max_* (Figure 5A, in gray) was 3.36 kHz. The upward half-track gave a lower ASSR *f_max_*; the bias between the two half-tracks was 0.27 kHz and the LoAs were 3.69 and −3.14 kHz.

The within-session repeatability of the ASSR amplitudes between the upward and downward half-tracks in response to AM2 alone provides a measure of the reliability of the recordings (Figure 5B, in red). The man ASSR amplitudes were 32.46 and 29.68 nV for the upward and downward half-tracks, respectively. The coefficient of repeatability was 20 nV, with a 2.78 nV bias relative to the two half-tracks, and the lower and upper LoAs were −17.59 and 23.15 nV, respectively. Since the mean amplitude of the two half-tracks was 32.45 nV, the 20 nV coefficient of repeatability was equivalent to 61.7% of variability. The ASSR amplitude variability between and within participants seems to be a major roadblock to using masked ASSRs for DR detection.

## Discussion

The purpose of the present study was to develop an objective method for diagnosing cochlear DRs using ASSRs and to compare the proposed method to a psychophysical measure. The method was tested using normally hearing participants. The psychophysical task was aimed at measuring the level of full and notched TEN needed to discriminate AM2 from pure tone and finding notch position at which the level of notched TEN needed to discriminate AM2 from pure tone was highest (psychophysical f_max_). The ASSR task was aimed at evaluating the differences in ASSR amplitudes in response to the full and notched TENs, and finding the notch position at which the ASSR amplitude was highest (ASSR *f_max_*). Since the data were collected using normally hearing adults, the psychophysical *f_max_* and ASSR *f_max_* were expected to be close to the AM2 frequency (2 kHz). It was also expected that each participant would have *f_max_* at similar notch position for the two techniques. However, this turned out not to be the case.

### Psychophysical Tasks

We obtained AM2MLs for low and high signal levels. The low signal level was 10 dB SL, the same level as used by Markessis et al. (2009) and suggested by Oxenham and Shera (2003) to obtain sharp tuning. We measured AM2ML as a function of the notch position in the TEN rather than as a function of notch width. The AM2ML maxima were clearly around the 2 kHz, namely when the notch was centered at 1.41, 2, or 2.82 kHz (as predicted using the excitation-pattern model; Moore et al., 1997). This probably occurred because the 2-octave-wide notch included the signal frequency for these three notch positions. The AM2ML was also measured at 60 dB SPL, the same as for the ASSR recordings, to allow a direct comparison. Since the auditory filters become broader with increasing level, the AM2ML curves for the high-level signal were expected to be less sharply tuned than those for the low-level signal. Indeed, for the high-level signal, the AM2ML curves were broader than for the low-level signal, which prevented accurate estimation of *f_max_* for the former.

### ASSR

We expected the maximum ASSR amplitude with CF_NOTCH_ at/or near the signal frequency. However, the predicted masking pattern was found only for 4 out of 15 participants (p02, p05, p13, and p14). It is possible that given our unique signal-masker combination (the AM2 carrier frequency of 2 kHz, 75 dB SPL broad-band TEN masker with a moving notch—hence both below and above the AM2 signal frequency), the measured ASSR may have been influenced by both excitatory masking and suppression (Delgutte, 1990; Gifford & Bacon, 2000; Oxenham & Plack, 1998), and the balance between the two may have varied across notch positions and across participants.

Furthermore, Foster et al. (2013) showed that participants had different optimal modulation rates that evoke largest ASSR. It is possible that by using the same ASSR modulation rate for all participants we recorded largest possible ASSRs only for a few of our participants whose “optimal” modulation rate was 87 Hz. This could have contributed to ASSR variability.

### Agreement Between Psychophysical and ASSR Data

Few studies compared psychophysical and electrophysiological masked AM tasks (e.g., Strelcyk et al., 2009). In the current study, the agreement between psychophysical and electrophysiological results was assessed via the Bland-Altman method (Bland & Altman, 1986). General trends in the psychophysical tasks were similar across participants at the low level. However, the ASSR amplitudes showed strong individual differences and only four participants showed the expected pattern. The variability of two octaves between the two methods is not clinically acceptable. The large width of the notch resulted in broad masking curves, making it impossible to estimate one distinct *f_max_* per masking curve, but a narrower notch might not elicit a clear ASSR using the SNR of −15 dB, as in the current study. A larger SNR might elicit a clear ASSR with a narrower notch, and this should be investigated systematically in future research.

### ASSR Repeatability

Poor repeatability of ASSR *f_max_* was found between the two mirrored half-tracks; the coefficient of repeatability was 3.36 kHz. Wilding et al. (2011) found a coefficient of repeatability for ASSR Response Amplitude Curve tip frequency between repeated recordings to be 0.39 kHz. The high repeatability coefficient of ASSR *f_max_* in our study may be due to a number of reasons. First, we compared ASSR *f_max_* between upward and downward half-tracks, instead of in two separate sessions with full (i.e., upward and downward) tracks. It is possible that the direction of CF_NOTCH_ change affected the values of ASSR *f_max_*. An effect of sweep direction on the position of the tip frequency was shown previously for fast PTCs: there was a systematic shift of the tip frequency in the direction of the sweep (Kluk & Moore, 2006b; Sęk et al., 2005; Sęk & Moore, 2011). A similar effect, but of different origin, may be present in our ASSR method as the majority of participants had higher *f_max_* for the upward half-track than for the downward half-track.

Most studies that examined the test–retest reliability of ASSR amplitudes used ASSRs to determine hearing thresholds. In one condition here, the AM2 signal was presented at 60 dB SPL alone, eliciting a mean ASSR amplitude of 32.4 nV and the coefficient of repeatability for upward and downward tracks, was 20 nV. Hence, the variability was about 62% of the mean amplitude. D’haenens et al. (2008) and Wilding et al. (2012) determined the repeatability of the ASSR amplitude between sessions for a 2 kHz carrier modulated at ∼ 80 Hz and using a signal level comparable to ours. D’haenens et al. expressed repeatability as twice the standard error of measurement (SEM), which is the standard deviation of the test–retest difference divided by √2. The mean amplitudes (± 2 SEM) were 52 nV (± 15) for a stimulus level of 50 dB HL. Hence, the variability was about 29% of the mean. Wilding et al. (2012) also recorded the ASSR at 50 dB HL, which corresponded to levels ranging from 52 to 72 dB SPL. Their coefficient of repeatability, estimated in the same way as here, was 29 nV. The mean amplitude being 73 nV, the variability was 40% of the mean. The aforementioned studies found both higher amplitudes and better repeatability of the ASSR than us. Differently to the current study, D’haenens et al. (2008) and Wilding et al. (2012) used online artifact rejection, so their analyses were based only on “clean” ASSRs. Also, our mean amplitude was lower than in the abovementioned studies, perhaps because we used a shorter continuous recording time for AM2 alone, which was 1 min every 9 min, giving 8 min of ASSR data in total (compared to 4 min continuous recording in Wilding et al. (2012) and 8 min continuous recording in D’haenens et al. (2008)). Our short and intermittent recording time may have limited the precision of the ASSR to AM2 because longer (and continuous) recording times have been shown to produce more precise threshold estimates of ASSR amplitudes (Luts & Wouters, 2004; Picton et al., 2005).

### F-Test for Hidden Periodicity and Hotelling's T2

Two metrics were used to assess the presence of the ASSR: the F-test for hidden periodicity and Hotelling’s T2. Valdes et al. (1997) showed that the sensitivity and specificity of the metrics were equivalent when detecting 80 Hz ASSRs (as used here). Of 125 ASSR amplitudes evaluated here using a significance level of 0.01, 106 had the same outcomes for the two tests: 60 were significant and 46 were not. Disagreement between the tests occurred for 19 ASSR amplitudes, which were significant only for the F-test. For participant p06, the F-test was significant for five notch positions and Hotelling’s T2 for none. Of the 19 ASSR amplitudes that were significant only for the F-test, five were below the noise floor (e.g., participant p15 in the full TEN condition). It is not possible to determine whether these 19 ASSR amplitudes were true or false positives, so the sensitivity and specificity of the metrics cannot be calculated. However, the results of participants p06 and p15, whose ASSR amplitudes were below the noise floor, suggest that these 19 ASSR amplitudes (or, at least 5 of them) were false positives for the F-test. If so, the F-test may have lower specificity than Hotelling’s T2.

### Low-Pass and High-Pass Filtered Maskers

**Figure 6.**
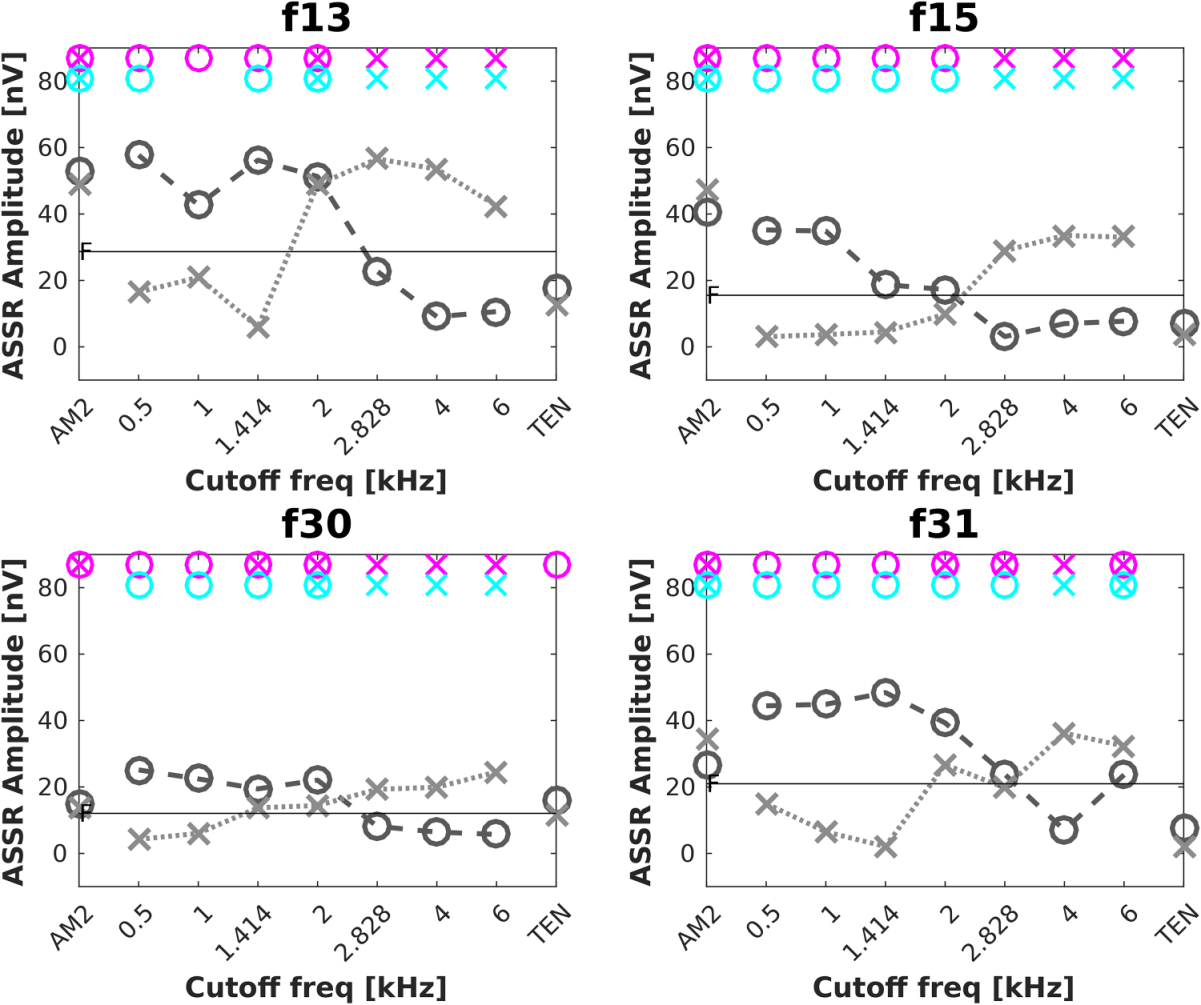
Individual ASSRs in responses to low-pass filtered TEN (dark gray circles and dashed lines) and high-pass filtered TEN (light gray crosses and dotted lines) for each participant. The horizontal solid line shows the EEG noise floor (F). The magenta and cyan circles and/or crosses indicate significant responses (*p* < .01) via the F-test for hidden periodicity and Hotelling’s T2 test, respectively. ASSR: auditory steady-state response; EEG: electroencephalogram; TEN: threshold equalizing noise.

Herdman et al. (2002) recorded ASSRs to AM signals alone and in a series of high-pass noise maskers with different cut-off frequencies. The derived-band ASSRs were defined as the ASSR amplitude difference between two ASSRs recorded in noise maskers with cut-off frequencies one octave apart. This approach might be applicable for diagnosing DRs at high frequencies but would not detect low-frequency DRs. Therefore, a method with both low-pass and high-pass filtered noise might be desirable. We investigated this idea by applying low-pass and high-pass filters to the TEN at 0.5, 1, 1.41, 2, 2.82, 4, and 6 kHz. ASSRs were recorded for four participants, p13 and p15, and two new participants (colleagues, a male and a female, 39 and 38 years old). The ASSR amplitudes evoked by the AM2 presented simultaneously with the filtered TEN are shown in Figure 6. For the low-pass filtered TEN (dark gray circles and dashed lines), the ASSR amplitudes were significantly above the noise floor for cut-off frequencies up to 2 kHz. For the high-pass filtered TEN (light gray crosses and dotted lines), the ASSR amplitudes were significantly above the noise floor for cut-off frequencies ≥2 kHz. Although only four participants were tested, the pattern of the ASSRs for low-pass and high-pass filtered TEN seems more reliable (at least for these participants) and predictable than for notched TEN. Therefore, this paradigm deserves further study.

## Conclusions

The aim of this study was to develop a new ASSR-based method that could be used for diagnosing DRs. The approach taken here was opposite to the one proposed by Markessis et al. (2009) and Wilding et al. (2011), whose methods relied on identification of the center frequency of a narrow-band noise masker giving the smallest ASSR amplitude. Instead, in our method we searched for the frequency (*f*_max_) giving the largest ASSR amplitude. To achieve this, ASSRs were elicited by an exponentially AM signal presented with the TEN that was band-stop filtered to give a notch with various center frequencies. A similar psychoacoustic task was used for comparison. Poor agreement between the psychophysical *f*_max_ (obtained for the low signal level) and ASSR *f*_max_ was observed, because of individual variability in the ASSR and the different processes measured by the psychophysical task and the ASSR. The proposed method needs further refinement, in particular to reduce individual variability in the ASSR. This could be achieved by decreasing the signal level to increase sharpness of the AM2ML curves, increasing the ASSR recording time to reduce ASSR amplitude variability, and implementation of online artifact rejection to reduce variability of the ASSR amplitude.

## Notes

1. p01 was not tested with the notch at 6 kHz, as we added this later as an additional condition.
